# Small heterodimer partner-interacting leucine zipper protein suppresses pain and cartilage destruction in an osteoarthritis model by modulating the AMPK/STAT3 signaling pathway

**DOI:** 10.1186/s13075-024-03417-3

**Published:** 2024-11-12

**Authors:** Jeonghyeon Moon, Keun-Hyung Cho, JooYeon Jhun, JeongWon Choi, Hyun-Sik Na, Jeong Su Lee, Seung Yoon Lee, Jun-Ki Min, Anan Shetty, Sung-Hwan Park, Seok Jung Kim, Mi-La Cho

**Affiliations:** 1 Departments of Immunobiology and Neurology, Yale School of Medicine, New Haven, CT USA; 2 Lab of Translational ImmunoMedicine (LaTIM), Catholic Research Institute of Medical Science, College of Medicine, The Catholic University of Korea, Seoul, 06591 Korea; 3 Department of Pathology, College of Medicine, The Catholic University of Korea, Seoul, 06591 Korea; 4 Department of Internal Medicine, and the Clinical Medicine Research Institute of Bucheon St. Mary’s Hospital, The Catholic University of Korea, Bucheon si, Gyeonggi-do Korea; 5 Institute of Medical Sciences, Canterbury Christ Church University, Medway Campus, Chatham, Kent UK; 6 Department of Internal Medicine, Division of Rheumatology, College of Medicine, Seoul St. Mary’s Hospital, The Catholic University of Korea, 222, Banpo‑daero, Seocho‑gu Seoul, 06591 Korea; 7 Department of Orthopaedic Surgery, Uijeongbu St. Mary’s Hospital, College of Medicine, The Catholic University of Korea, Cheonbo-ro, Uijeongbu-si, Gyeonggi-do 271, Korea

**Keywords:** Osteoarthritis (OA), Small heterodimer partner-interacting leucine zipper protein (SMILE), Cyclic AMP-response element binding protein Zhangfei (CREBZF), Signal transducer and activator of transcription 3 (STAT3), Helper T cell (th cell)

## Abstract

**Objective:**

Osteoarthritis (OA) is a degenerative joint disease caused by the breakdown of joint cartilage and adjacent bone. Joint injury, being overweight, differences in leg length, high levels of joint stress, abnormal joint or limb development, and inherited factors have been implicated in the etiology of OA. In addition to physical damage to the joint, a role for inflammatory processes has been identified as well. Small heterodimer partner-interacting leucine zipper protein (SMILE) regulates transcription and many cellular functions. Among the proteins activated by SMILE is the peroxisome proliferator-activated receptor (PPAR) γ, which mediates the activities of CD4 + T helper cells, including Th1, Th2, and Th17, as well as Treg cells. PPAR-γ binds to STAT3 to inhibit its transcription, thereby suppressing the expression of the NF-κB pathway, and in turn, the expression of the inflammatory cytokines interferon (IFN), interleukin (IL)-1β, IL-6, and tumor necrosis factor (TNF)-α, which are sub-signals of STAT3 and NF-κB.

**Methods:**

OA was induced in control C57BL/6 mice and in C57BL/6-derived SMILE-overexpressing transgenic (SMILE Tg) mice. The protein expression levels in the joint and spleen tissues were analyzed by immunohistochemistry and immunofluorescence images. In addition, flow cytometry was performed for detecting changes of the changes of immune cells.

**Results:**

Less cartilage damage and significantly reduced levels of OA biomarkers (MMP13, TIMP3 and MCP-1) were observed in SMILE Tg mice. Immunohistochemistry performed to identify the signaling pathway involved in the link between SMILE expression and OA revealed decreased levels of IL-1β, IL-6, TNF-α, and phosphorylated AMPK in synovial tissues as well as a significant decrease in phosphorylated STAT3 in both cartilage and synovium. Changes in systemic immune cells were investigated via flow cytometry to analyze splenocytes isolated from control and SMILE Tg mice. SMILE Tg mice had elevated proportions of CD4 + IL-4 + cells (Th2) and CD4 + CD25 + Foxp3 + cells (Treg) and a notable decrease in CD4 + IL-17 + cells (Th17).

**Conclusion:**

Our results show that overexpressed SMILE attenuates the symptoms of OA, by increasing AMPK signaling and decreasing STAT3, thus reducing the levels of inflammatory immune cells.

## Introduction

Osteoarthritis (OA) is a degenerative joint disease caused by the breakdown of joint cartilage and adjacent bone [1, 2]. The most common symptoms of OA are pain, stiffness, and swelling of the joint resulting in a decreased range of motion and weakness of the involved limb [3, 4]. The symptoms are usually slowly progressive [5], and while they do not significantly affect longevity, they do critically compromise quality of life [6].

Among the causes of OA are joint injury, being overweight, differences in leg length, high-level joint stress, abnormal joint or limb development, and inherited factors [7], resulting in physical damage to the joint and the induction of inflammatory processes [8–10]. The exercise for weight loss, the use of a cane for decreasing joint stress, and pain medications [11], mainly paracetamol (acetaminophen), naproxen, and ibuprofen [12–14] were being used to treat OA. Arthroplasty is performed in patients with severe, persisting OA [15]. However, the long-term use of anti-inflammatory drugs is problematic and an artificial joint is maintained only for 10–15 years [16]. Novel, alternative approaches to the treatment of OA are therefore needed.

Physical joint damage and joint inflammation recruit immune cells [17] to the synovium, where they release interleukin (IL)-1β, IL-6, and tumor necrosis factor (TNF)-α [18, 19]. In addition, activated macrophage release monocyte chemoattractant protein (MCP)-1 [20], which induces chondrocytes in cartilage to express matrix metalloproteinases (MMPs) and tissue inhibitors of metalloproteinases (TIMPs), responsible for joint destruction [21, 22]. Here we show that OA can be ameliorated by modulating immune cells and their activities.

Small heterodimer partner-interacting leucine zipper protein (SMILE) is an isoform of cyclic AMP-response element binding protein Zhangfei (CREBZF) [23, 24], which makes up a family of activating transcription factor/cAMP response element-binding proteins (ATF/CREB) that regulate various cellular functions, including transcription [25]. SMILE acts as a transcription factor and an insulin-inducible corepressor that decreases peroxisome proliferator-activated receptor (PPAR)-γ expression, hepatic glucogenesis, and the adenosine monophosphate-activated kinase (AMPK) signaling pathway [26]. PPAR-γ mediates the activities of CD4 + T helper cells, including Th1, Th2, and Th17 cells, and Treg cells [27, 28]. It also binds to signal transducer and activator of transcription 3 (STAT3) to inhibit its transcription and suppresses the nuclear factor-κB (NF-κB) pathway [29], in turn downregulating expression of the inflammatory cytokines interferon (IFN), IL-1β, IL-6, and TNF-α, which are sub-signals of STAT3 and NF-κB [30]. AMPK prevents the activation of mammalian target of rapamycin (mTOR), which regulates T cell differentiation and inhibits Th17 cell activation through mTOR and STAT3 inhibition [31–34]. AMPK also plays a critical role in osteoclastogenesis [35, 36].

In this study, a mouse model of OA was established in both transgenic mice overexpressing SMILE and in non-transfected control mice to investigate whether SMILE overexpression has a histologically demonstrable protective function on OA progression and is able to suppress inflammation by regulating the expression of immune cells and cytokines. Our results suggest that the therapeutic targeting of SMILE can prevent OA progression, by suppressing the excessive activation of immune cells.

## Materials and methods

### Animals

Seven-week-old male C57BL/6 mice weighing 18–23 g were purchased from Orient Bio (Korea). The animals were bred and managed in a specific-pathogen-free environment at the Laboratory Animal Laboratory of the College of Medicine, Catholic University of Korea. They were fed gamma-ray-sterilized standard mouse feed (Ralston Purina, USA) and provided with water subjected to high-pressure sterilization through an R/O water treatment autoclave. Five mice were kept in each feeding and breeding space under temperature- and light-controlled conditions (21–22 °C, 12/12 h light/dark cycle).

SMILE transgenic mouse were obtained by microinjecting C57BL/6 mice with a pcDNA3.0 vector containing the cytomegalovirus promoter and the SMILE gene fragment (GenScript Corp., USA) with codon optimization for expression in mammalian cells. C57BL/6 mice and SMILE Tg mice were crossed for 10 generations, and the presence of the transgene was confirmed through PCR of DNA extracted from the tail.

Experimental procedures were conducted in accordance with the Laboratory Animal Welfare Act, Laboratory Animal Care Guidelines; the rodent experimentation guidelines and policies of the Institutional Animal Care and Use Committee (IACUC) of the College of Medicine, the International Council for Laboratory Animal Science, and the Ministry of Food and Drug Safety. The absence of specific pathogens was monitored (IACUC approval numbers: 2020-0306-02 and 2021-0010-01).

### Creation of the mouse OA model through monosodium iodoacetate (MIA) intra-articular injection

Osteoarthritis was induced through a single intra-articular injection of MIA. After anesthesia with 2% isoflurane, OA was induced via the intra-articular injection of 0.6 mg MIA/kg (total 20 µL volume into the right knee via a Hamilton syringe) into the right knee joint of mice [37]. From the day of injection, the pain threshold was measured once a week for 5 weeks as described below.

### Pain threshold assessment

An electronic version of the von Frey aesthesiometer (IITC, USA) was used to evaluate the sensitivity to joint pain in blind tests. A load was applied by placing the filament vertically on the plantar surface, and the automatically measured weight was recorded based on when the mouse retracted its foot from the filament. The maximum applied force was 90 g.

### Histopathological analysis

After 5 weeks, the knee joint of each mouse was collected and fixed with 10% formalin (Sigma-Aldrich, USA). The fixed knee joint tissues were subsequently decalcified in Calci-clear (National Diagnostics, United States), embedded in paraffin, and serially sectioned into slices of 5 μm thickness. The tissue section samples were de-paraffinized in xylene, dehydrated in an alcohol series, and stained with Safranin O. The stained knee joint tissue was evaluated based on the OARSI index and the total Mankin score. Cartilage damage was scored as described previously [38, 39].

### Immunohistochemistry

To analyze pathological changes of joint tissue, the cartilage and synovial tissues of medial joint were sectioned by coronal plane. Formalin-fixed, paraffin-embedded tissue sections were treated with proteinase K in Tris-ethylenediaminetetraacetic acid buffer for antigen retrieval and washed in 1× phosphate-buffered saline (PBS; pH 7.5). Endogenous peroxidase activity was removed by treatment with methanol and 3% H_2_O_2_. The sections were stained with anti-MMP13 and anti-TIMP3, as anabolic and catabolic markers, and anti-IL-1β (NB600-633, NOVUS), anti-IL-6(NB600-1131, NOVUS), anti-TNF-α (ab6671, Abcam), anti-MCP-1(ab7202, Abcam), anti-IL-10(BS-20373, Bioss), and anti-pSTAT3 ser727 (ab30647, Abcam), as inflammatory and anti-inflammatory markers. Anti-SMILE (NBP2-57754, NOVUS) and anti-p-AMPK (44-1150G, Invitrogen) antibodies were used to confirm SMILE and AMPK expression in SMILE Tg mice and control mice. The sections were incubated in the antibody overnight at 4 °C followed by incubation with horseradish peroxidase-conjugated secondary antibody for 30 min. The final color product was developed using 3,3-diaminobenzidine (Dako, Denmark). Sections were counterstained with Mayer’s hematoxylin and examined under a microscope (Olympus, Japan).

### Immunofluorescence staining

The tissue sections were prepared as described for immunohistochemistry (IHC) and after antigen retrieval using proteinase K were washed with 1× PBS. Then they were blocked for 30 min in 10% normal goat serum (NGS) diluted in 1× PBS, incubated overnight at 4 °C in anti-RIPK3 (PA5-19956, Thermo Fisher), anti-p-MLKL (ab196436, Abcam), anti-CD4 (ab200594, Abcam), and AF647-conjugated IL-17 (sc-374218 AF647, Santa Cruz) diluted in 10% NGS, washed with 1× PBS, and incubated at room temperature for 2 h with the following secondary antibodies: for RIPK3 and p-MLKL, APC-conjugated anti-rabbit antibodies (A-10931, Invitrogen), and for CD4 AF488-conjugated anti-rat antibody (A-11006, Invitrogen). The immunostained sections were washed, nuclear stained using 4’,6-diamidino-2-phenylindole (DAPI; D3571, Invitrogen), and mounted. Fluorescence imaging and imaging analysis were performed using a confocal microscope (LSM 900; ZEISS), and ZEN2012 software (blue edition; ZEISS).

### Flow cytometry

Splenocytes were collected from the euthanized mice and transferred to a 24-well plate. Populations of Th1, Th2, Th17, and Foxp3-positive Treg cells were identified by stimulating the cells for 4 h with 25 ng PMA/mL and 250 ng ionomycin/mL (both from Sigma-Aldrich, St. Louis, MO, USA) and Golgi Stop (BD Biosciences, San Diego, CA, USA). The stimulated cells were stained with Percp-conjugated anti-CD4 antibody (eBiosciences), fixed and permeabilized using the Cytofix/Cytoperm Plus kit (BD Biosciences), in accordance with the manufacturer’s instructions, and stained with PE-conjugated anti-IL-4, APC-conjugated anti- IFN-γ, and FITC-conjugated anti-IL-17 (eBioscience) antibodies. Treg cells were analyzed by labeling splenocyte surface proteins with Percp-conjugated anti-CD4 and APC-conjugated anti-CD25 antibodies, followed by fixation, permeabilization, and intracellular staining with PE-conjugated anti-Foxp3 antibodies, in accordance with the manufacturer’s protocol. All samples were examined using a FACS Calibur device (BD Pharmingen).

### Statistical analysis

The data are presented as the mean ± SD of at least three independent experiments or at least three independent samples. In vitro experiments were independently repeated three or more times, and each experiment had at least three samples. One-way analysis of variance followed by Bonferroni’s post hoc test was used to compare differences among three or more groups. The Mann-Whitney U test was used to compare numerical data between groups. The Shapiro-Wilk test and Levene’s test were used to assess the Gaussian distribution and equality of variance, respectively. A P value < 0.05 was considered statistically significant. All statistical analyses were performed with ANOVA for Mac.

## Results

### SMILE overexpression suppresses pain and the destruction of articular cartilage in MIA-induced OA in mice

**Fig. 1:**
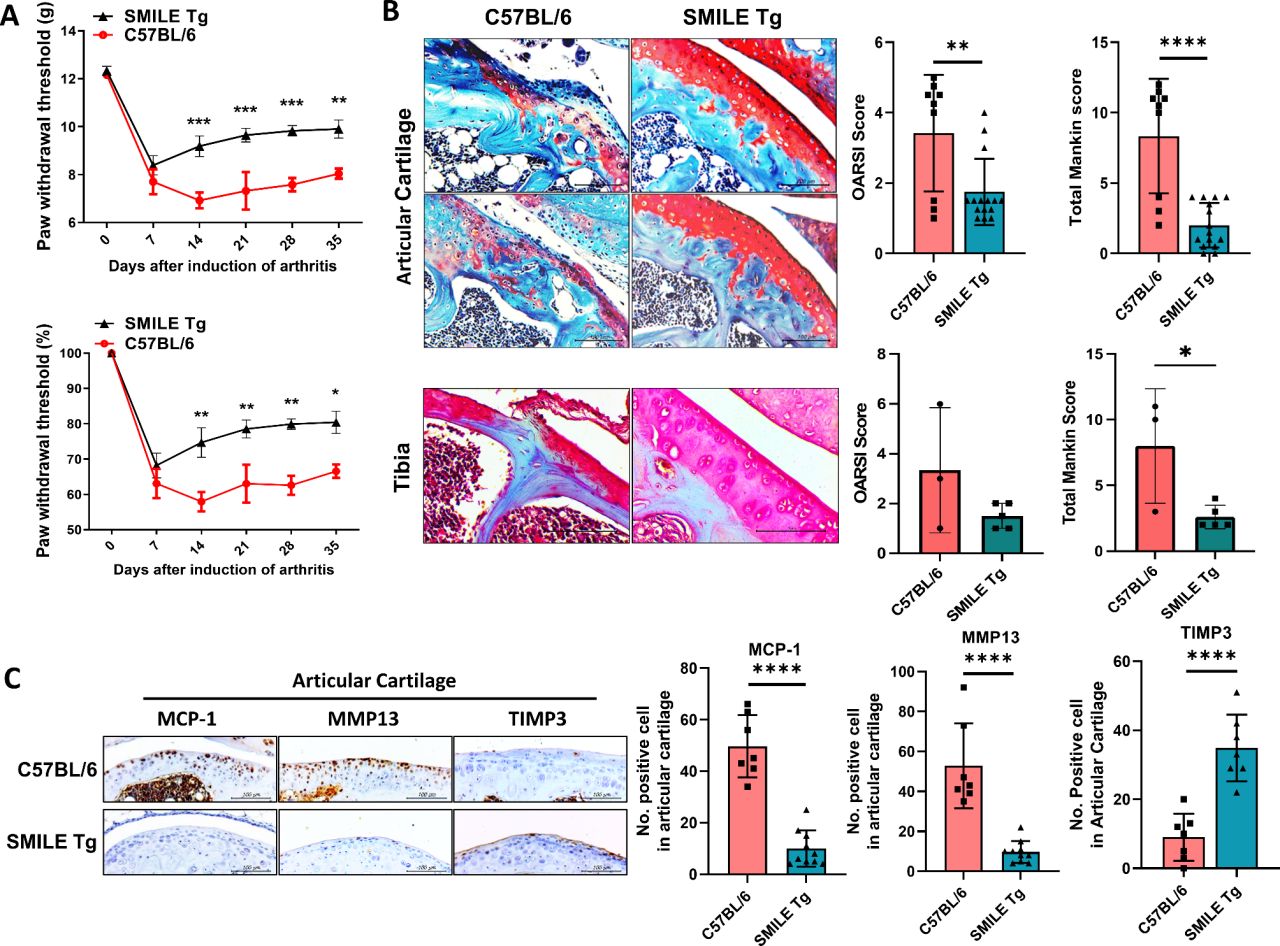
Upregulation of SMILE inhibits the progression of OA and cartilage damage in MIA-induced OA in mice. (**A**) Joint pain was analyzed as the difference between the PWL (left) and PWT (right) in control C57BL/6 MIA-induced OA mice (C57BL/6) and SMILE overexpression vector injected transgenic mice (SMILE Tg). The mice were evaluated for 5 weeks. Nociceptive testing was performed using a dynamic plantar esthesiometer, which is an automated version of the von Frey hair assessment tool. The threshold for pain was shown as weight (g) and relative weight load (%) based on Day 0 as 100%. (**B**) Safranin O staining of the articular cartilage and tibia tissues of C57BL/6 mice and SMILE Tg. Bar graphs show the average OARSI and Mankin scores. (**C**) Immunohistochemistry was performed to assess the expression of MCP-1, MMP13 and TIMP3 in articular cartilage of C57BL/6 and SMILE Tg mice. The data are presented as the mean ± standard deviation. Scale bars = 100 μm (**B** and **C**). *p < 0.05, **p < 0.01, ***p < 0.001, and ****p < 0.0001

To investigate the pain-killing capacity of SMILE overexpression, SMILE Tg mice were established from C57BL/6 mice and OA was induced in both by MIA injection. Pain was monitored and quantified for 5 weeks using the electronic von Frey system, and weight bearing using a capacitance meter. The results showed that SMILE Tg mice had significantly less pain than control mice (Fig. 1A). Safranin O staining showed that SMILE overexpression inhibited the destruction of articular cartilage and tibia tissues, as assessed based on the OARSI and Mankin scores respectively (Fig. 1B). In SMILE Tg mice, the expression of MCP-1 and MMP13 notably decreased while that of TIMP3 significantly increased (Fig. 1C). These data demonstrate the therapeutic effect of SMILE overexpression on OA.

### Comparison of proinflammatory cytokines, OA-catabolic factors, and OA-anabolic factors in synovial tissue of control and SMILE Tg mice

**Fig. 2:**
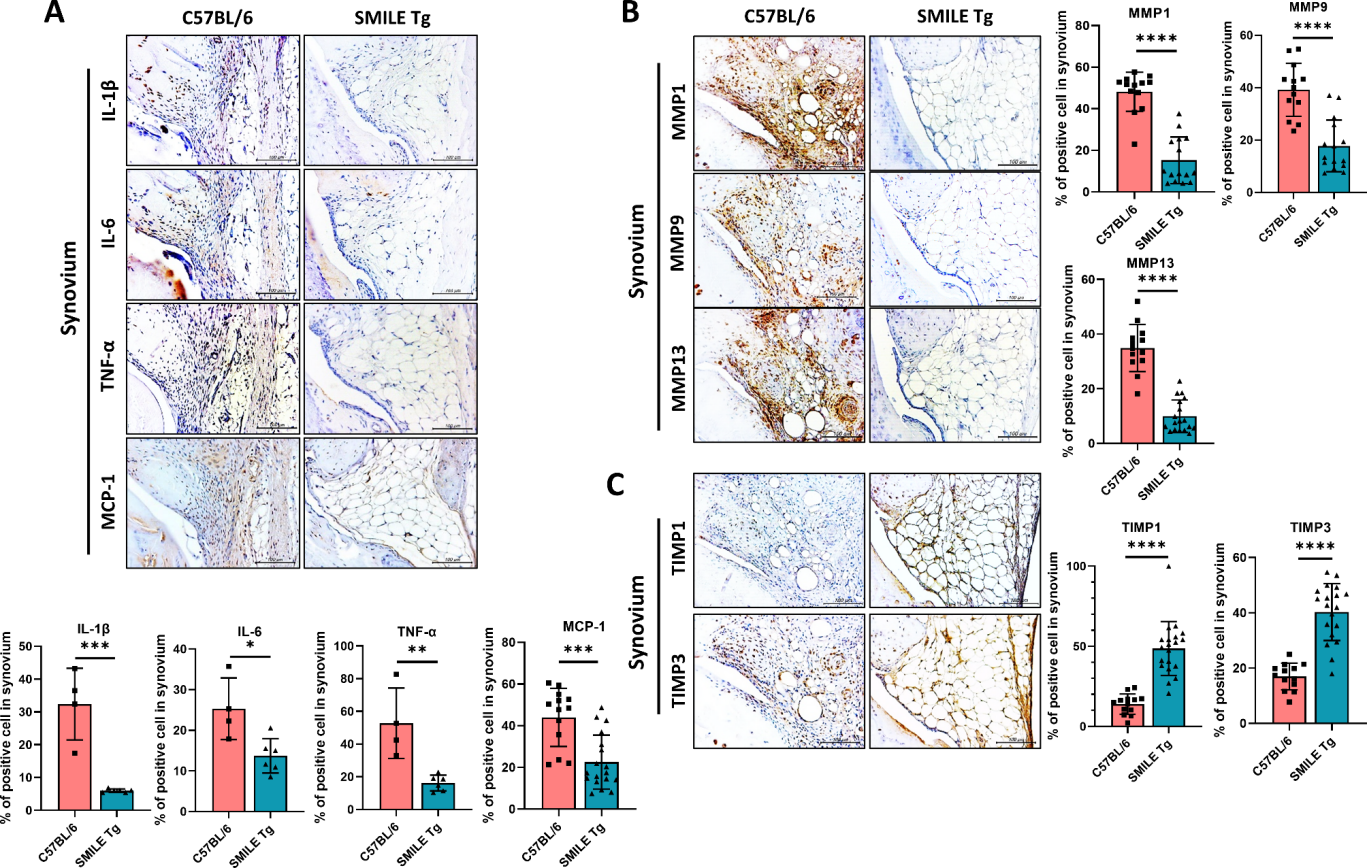
Levels of proinflammatory cytokines and OA-catabolic factors are decreased and those of OA-anabolic factors are increased in the synovium of SMILE Tg mice. Representative immunohistochemistry images of the synovium of control C57BL/6 and SMILE Tg mice showing the levels of (**A**) pro-inflammatory cytokines such as IL-1β, IL-6, TNF-α, and MCP-1, (**B**) OA-catabolic (MMP1, MMP9, and MMP13) and (**C**) OA-anabolic factors (TIMP1 and TIMP3). The data are presented as the mean ± standard deviation. Scale bars = 100 μm (A, **B** and **C**). *p < 0.05, **p < 0.01, ***p < 0.001, and ****p < 0.0001

Differences in chemokine and cytokine expression in the synovial tissue of control and SMILE Tg mice were examined by IHC. Expression of the proinflammatory cytokines IL-1β, IL-6, TNF-α, and MCP-1 was lower in SMILE Tg mice than in control mice (Fig. 2A). The expression of OA-related catabolic and anabolic factors was also examined using IHC, which showed decreases in the catabolic factors MMP1, MMP9, and MMP13 (Fig. 2B) and increases in the expression of the OA-anabolic factors TIMP1 and TIMP3 (Fig. 2C) in SMILE Tg mice. These data suggested that SMILE overexpression inhibits OA by regulating cytokine and chemokine expression.

### Changes in the AMPK and STAT3 signaling pathways induced by SMILE overexpression

**Fig. 3:**
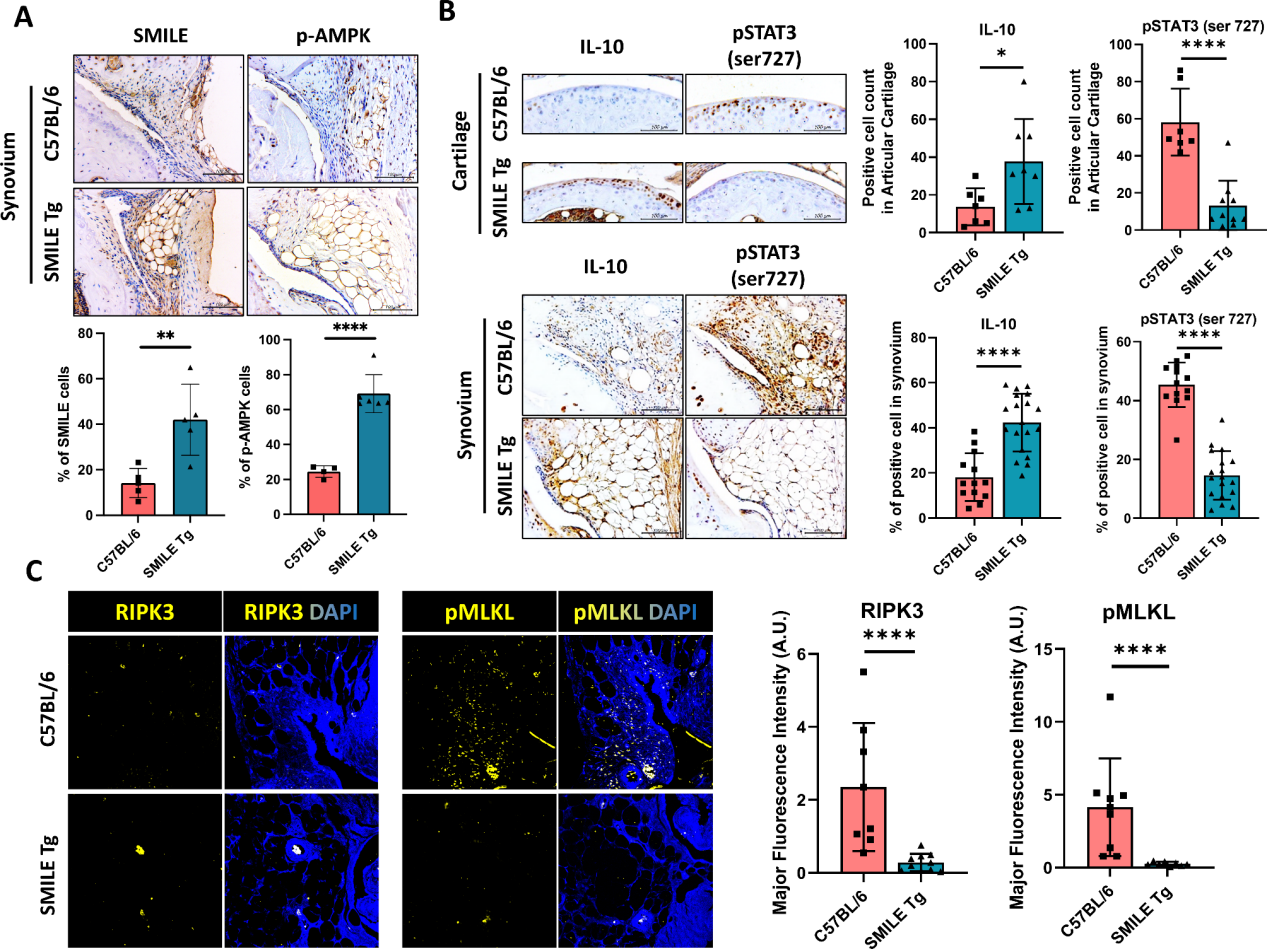
AMPK and STAT3 signaling pathways and necroptosis are significantly altered by SMILE overexpression. (**A**) Immunohistochemistry images show AMPK expression in the synovial tissues of C57BL/6 and SMILE Tg mice. (**B**) The expressions of IL-10 and phosphorylated STAT3 (pSTAT3) in the synovial and cartilage tissues of C57BL/6 and SMILE Tg mice. (**C**) Immunofluorescence shows the cell nuclei (DAPI; blue) and the expressions of RIPK3 (yellow) and phosphorylated MLKL (pMLKL; yellow) in synovial tissues of C57BL/6 and SMILE Tg. The data are presented as the mean ± standard deviation. Scale bars = 100 μm (**A** and **B**). *p < 0.05 and ****p < 0.0001

To identify the signaling pathway(s) in the SMILE-mediated inhibition of OA progression via the regulation of cytokine and chemokine expression, synovial and cartilage tissues were subjected to IHC and immunofluorescence staining. SMILE overexpression was confirmed in SMILE Tg mice (Fig. 3A; cartilage data not shown). In the latter, IHC also revealed an increase in phosphorylated AMPK (pAMPK) expression in synovial tissue (Fig. 3A) as well as an increase in IL-10 expression and a decrease in pSTAT3 expression in the cartilage and synovium (Fig. 3B). Immunofluorescence staining of the inflammatory cell death markers RIPK3 and phosphorylated MLKL (pMLKL) showed that the expression of both was significantly decreased in SMILE Tg mice (Fig. 3C). Taken together, these results show that SMILE expression activates AMPK signaling while suppressing STAT3 expression and inflammatory cell death.

### SMILE overexpression has systemic anti-inflammatory effects through the regulation of helper T cell frequency

**Fig. 4:**
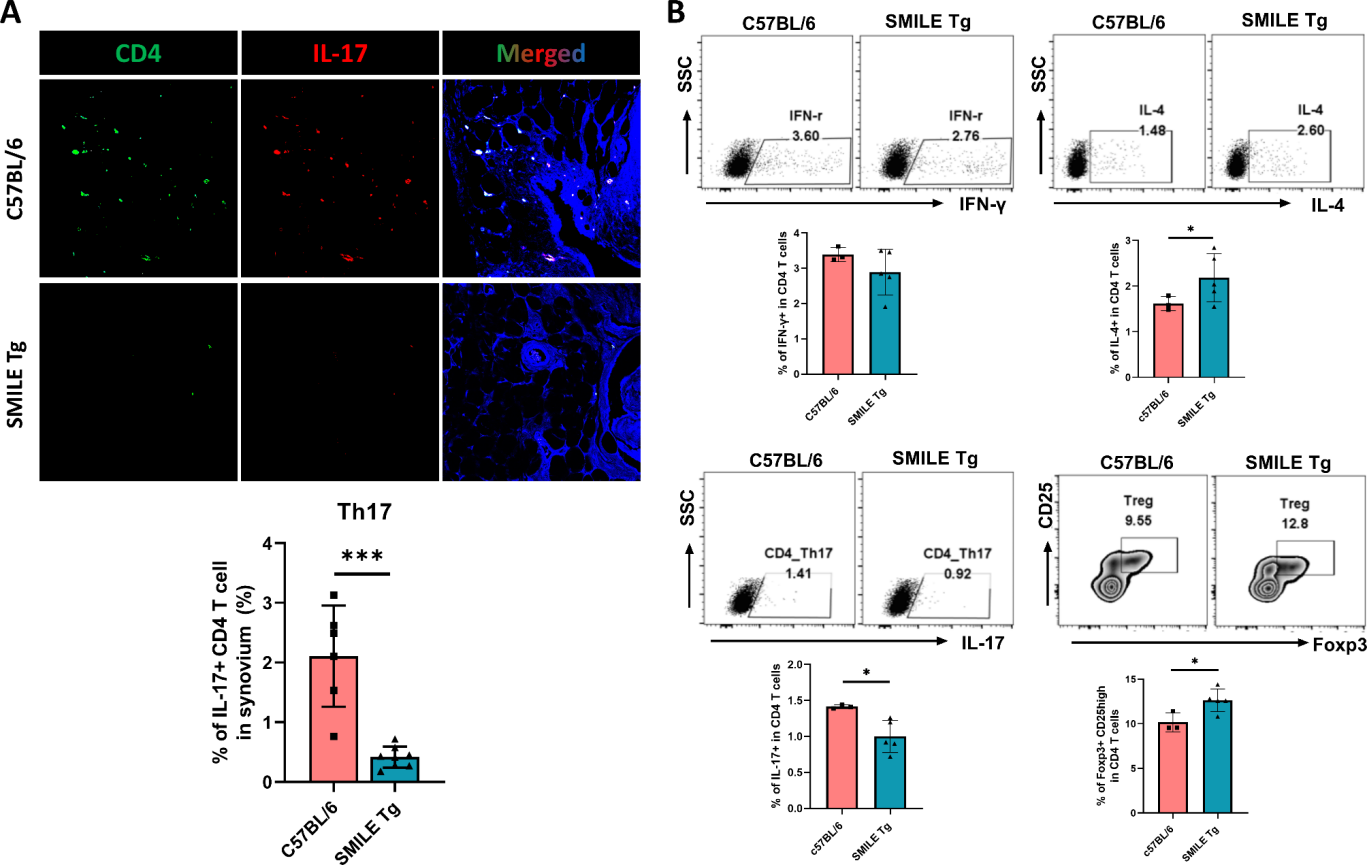
Frequency of changes in systemic helper T (Th) cells in SMILE Tg mice. (**A**) Representative immunofluorescence images show the spleen tissues of C57BL/6 and SMILE Tg stained with anti-CD4 antibody (green) and anti-IL-17 antibody (red). Nuclei were stained by DAPI (blue). (**B**) The splenocytes of C57BL/6 and SMILE Tg were analyzed by flow cytometry. Th1 (CD4^+^ IFNγ^+^), Th2 (CD4^+^ IL-4^+^), Th17 (CD4^+^ IL-17^+^) and regulatory T cell (Treg; CD4^+^ CD25^high^ Foxp3^+^) populations are shown. The data are presented as the mean ± standard deviation. *p < 0.05 and ***p < 0.001

The effect of SMILE overexpression on systemic immune cells was investigated by examining the frequency of helper T cell subtypes in spleen tissue. Th17 cells, which are critically involved in inflammation and arthritis, were identified in spleen tissues by immunofluorescence. The results showed a significant decrease in Th17 cells (CD4^+^ IL-17^+^) in SMILE Tg mice (Fig. 4A). An analysis of splenocytes by flow cytometry showed a non-significant decrease in Th1 (CD4^+^ IFN-γ^+^), a significant decrease in Th17 cells, and substantial decreases in Th2 (CD4^+^ IL-4^+^) and Treg (CD4^+^ CD25^+^ Foxp3^+^) cells in SMILE Tg mice (Fig. 4B). Thus, SMILE overexpression regulates both OA progression and systemic immune cell differentiation through the AMPK/STAT3 signaling pathway.

## Discussion

The etiology of OA is complex and multifactorial, including age, physical stress, genetic, biological, biomechanical, and joint-specific factors [1]. Joint replacement is effective treatment for symptomatic end-stage OA patients, but the functional outcomes can be poor and the lifespan of the prosthesis is limited [1, 40]. Consequently, the therapeutic focus in OA is shifting to the prevention and treatment of early-stage disease.

Both AMPK and STAT3 have been well studied [41, 42]. AMPK is a crucial regulator of energy metabolic homeostasis [43, 44] and AMPK signaling inhibits the inflammatory response by regulating the NF-κB system [45]. STAT3 regulates the transcription of pro-inflammatory cytokines and thus triggers an inflammatory response [30, 46]. We previously investigated the SMILE-mediated regulation of AMPK and STAT3 in autoimmune diseases using animal models. In a mouse model of rheumatoid arthritis induced by ursodeoxycholic acid, the overexpression of AMPK and p38 in CD4 + T cells inhibited Th17 differentiation by naive T cells, thereby suppressing the inflammatory response [47]. In addition, we showed a significant decrease in STAT3 expression in mice transiently overexpressing SMILE following DNA vector injection and in SMILE Tg mice. In a mouse model of inflammatory bowel disease, obtained through oral administration of dextran sodium sulfate, STAT3 expression in mice transiently overexpressing SMILE and SMILE Tg mice was reduced, thus also reducing the abundance of pro-inflammatory cytokines such as IL-17 and inhibiting disease progression [48].

A similar approach was used to investigate the expression of AMPK and STAT3 in a mouse model of rheumatoid arthritis. In both types of SMILE-overexpressing mice, disease development was suppressed, evidenced by decreases in the expression of B-cell activating factor receptor (BAFF-R) and STAT3 and increases in the expression of AMPK, which led to the inhibition of B cell activation and the prevention of inflammation [49]. These studies demonstrated the therapeutic potential of SMILE overexpression—through its regulation of AMPK and SAT3 signaling—in autoimmune diseases. Based on those results, we investigated whether SMILE overexpression could ameliorate disease progression in OA by regulating AMPK and STAT3.

OA was induced by MIA injection into the joints of SMILE overexpressing and control mice. For the OA rodent model, the destabilization of the medial meniscus (DMM) surgical model and MIA model are generally used [50]. The DMM model has slow OA progress, similar to human OA, and has a structurally similar pathological phenotype after surgery [51]. On the other hand, MIA is accompanied by more severe inflammation and joint damage compared to DMM. We used MIA, a severe OA model, to inhibit the progression of aggressive OA by regulating the expression of the SMILE gene and to understand the mechanisms of OA and SMILE. SMILE Tg mice had significantly less joint pain than control mice while safranin O staining of articular cartilage showed that SMILE expression inhibited bone erosion. In addition, a decrease of the OA-catabolic factors MCP-1 and MMP13, an increase in the OA-anabolic factor TIMP3, an increase in IL-10, and a decrease in phosphorylated STAT3 in articular cartilage were demonstrated by IHC. In synovial tissues, IHC showed that SMILE overexpression decreased the levels of the pro-inflammatory cytokines IL-β, IL-6, TNF-α, and MCP-1, increased the levels of the anti-inflammatory cytokine IL-10, decreased the levels of OA-catabolic factors (MMP1, MMP9, and MMP13) and phosphorylated STAT3, and increased the levels of OA-anabolic factors (TIMP1 and TIMP3) and phosphorylated AMPK. Especially, we focused on the OA influence of the immune system. Therefore, we attempted to suggest a mechanism by which SMILE inhibits the progression of OA by showing differences in the expression of MMPs that promote the remodeling of extracellular matrix (ECM), and TIMP1 and TIMP3, which are known proteins that can inhibit these MMPs and suppress tissue inflammation.

Spleen tissue and splenocytes were stained to investigate the systemic immune response according to SMILE expression, which in SMILE Tg mice showed a reduced expression of inflammatory cell death markers, a decrease in Th17 cells in spleen tissue, a reduced proportion of Th17 cells in splenocytes, and increases in the populations of Th2 and Treg cells.

Taken together, our results demonstrate that the overexpression of SMILE attenuates OA progression by mechanisms that include the regulation of immune cells, the production of pro-inflammatory cytokines, regulation of the levels of OA-catabolic and OA-anabolic factors, activation of AMPK signaling, and repression of the STAT3 pathway.

Although the evidence supporting a role of chronic inflammation in OA is well-known, only recently have we begun to realize that this inflammation has a pivotal role in the pathogenesis of OA [52]. It is also clear that OA is a disease of the whole joint, affecting not only the articular cartilage and synovium but also the muscles, ligaments, subchondral bone, and immune cells. OA can perhaps also be considered a systemic disease, in which systemic inflammation interacts with the joint tissues [53, 54].

SMILE does not appear to be expressed specifically in chondrocytes or fibroblasts following as public data such as GTEx, BioGPS, Illumina Human BodyMap, and SAGE data set. SMILE is a transcription factor that is mainly present in the nucleus and mitochondria. Therefore, SMILE expression is expressed in all tissues of our body rather than depending on a specific tissue or cell type. It is a transcription factor that plays an important role and is mainly abundant in the nucleus and mitochondria [55, 56] and mitochondria is able to involve the OA progression [57]. Therefore, SMILE tends to be expressed more highly in mitochondria-rich tissues, liver, and immune cells [58, 59]. Thus, synovial tissue, which is rich in blood vessels, is likely to be more affected by systemic immune cells due to changes in SMILE expression. In fact, in our synovial site tissue data, mice in which OA was induced via MIA had a decrease in adipose tissue and an increase in fibroblasts, resulting in more severe synovial site immune cell infiltration and fibrosis [60, 61].

**Fig. 5:**
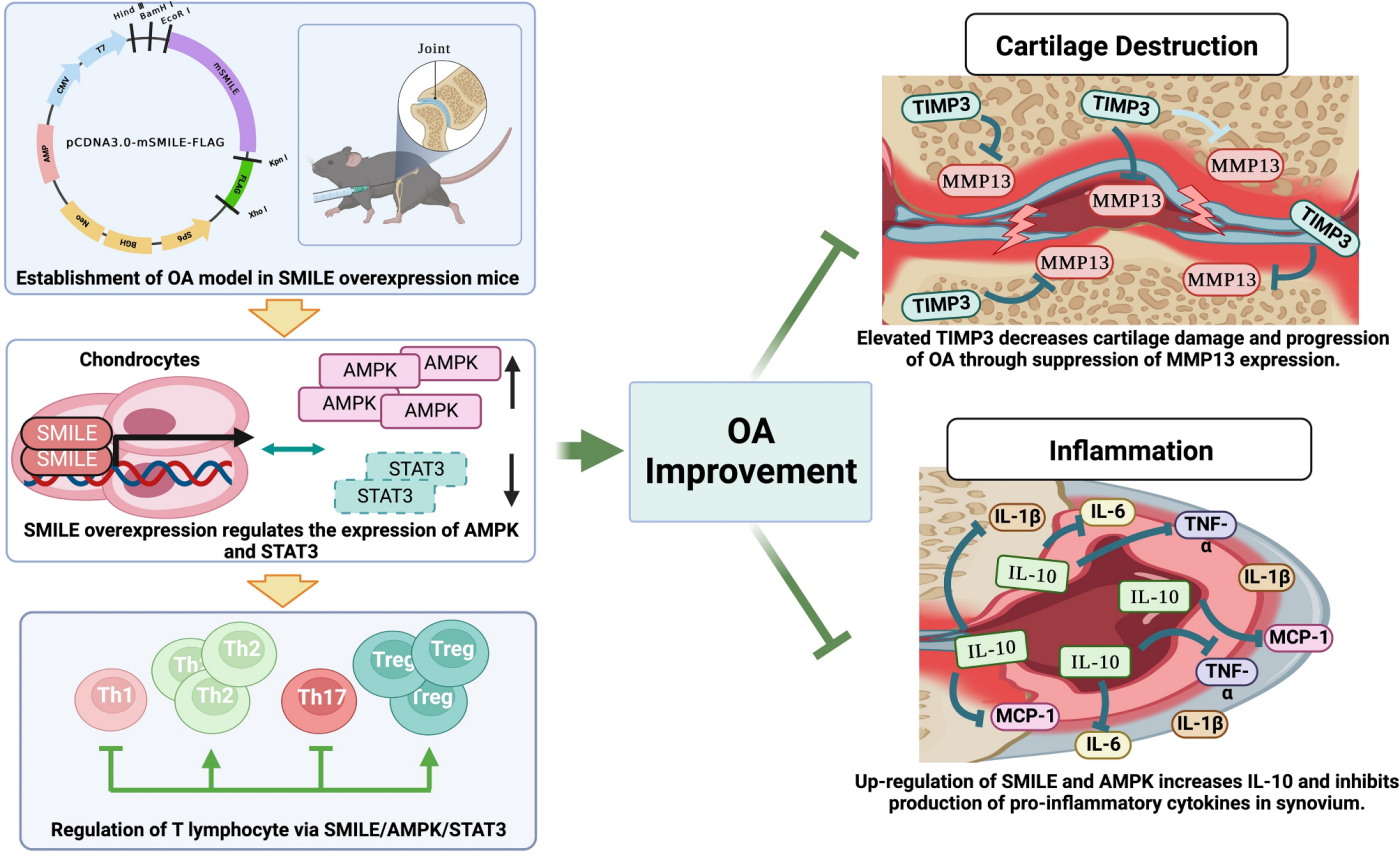
Schematic diagram of therapeutic effect of SMILE on OA through the regulation of AMPK and cytokine production. SMILE overexpression leads to an increase in AMPK and a decrease in STAT3 in joint tissue, in addition to changing the proportion of immune cell subsets. SMILE overexpression also suppresses both the expression of OA-catabolic factors and inflammatory cytokine production

Here, this study shows that SMILE expression can help suppress OA progression and symptoms related with systemic and topical inflammation using OA mouse model (Fig. 5). This may not be a complete solution to OA; however, it can help to better understand and treat the mechanism of OA beyond the fact that OA is simply a topical disease caused by physical stimulation. Taken together, this study based on a mouse model of OA shows that SMILE expression can help suppress OA progression and symptoms by intervening in both systemic and topical inflammation. As such, it suggests an approach to treat OA mechanistically rather than at the symptom level.

## Conclusion

Overall, this study shows the critical role of SMILE in osteoarthritis. SMILE overexpression showed the notable decreased damage of cartilage and bone in experimental osteoarthritis animal model. It leads to change of expression of STAT3/AMPK signaling pathway. SMILE overexpression regulates not only physical tissue damage, but also regulation of immune cell frequency. Hence, it suggests a novel approach to treat osteoarthritis via regulation of SMILE expression.

## Data Availability

No datasets were generated or analysed during the current study.

## Abbreviations

OA: Osteoarthritis
SMILE: Small Heterodimer Partner-Interacting Leucine Zipper Protein
PPAR: Peroxisome Proliferator-Activated Receptor
IFN: Interferon
TNF: Tumor Necrosis Factor
AMPK: 5’ Adenosine Monophosphate-Activated Protein Kinase
STAT3: Signal Transducer and Activator of Transcription 3
Th: helper T
IL: Interleukin
MMPs: Matrix Metalloproteinases
TIMPs: Tissue Inhibitors of Metalloproteinases
CREBZF: Cyclic AMP-Response Element Binding Protein Zhangfei
mTOR: Mammalian Target of Rapamycin
IHC: Immunohistochemistry
DAPI: 4’,6-diamidino-2-phenylindole
MIA: Monosodium Iodoacetate
PBS: Phosphate-Buffered Saline
BAFF-R: B-cell Activating Factor Receptor
